# Selenium Supplementation Improved Cardiac Functions by Suppressing DNMT2-Mediated *GPX1* Promoter DNA Methylation in AGE-Induced Heart Failure

**DOI:** 10.1155/2022/5402997

**Published:** 2022-04-06

**Authors:** Huolan Zhu, Xiang Wang, Xuyang Meng, Yiya Kong, Yi Li, Chenguang Yang, Ying Guo, Xiqiang Wang, Haini Yang, Zhongwei Liu, Fang Wang

**Affiliations:** ^1^Department of Geriatrics, Shaanxi Provincial Clinical Research Center for Geriatric Medicine, Shaanxi Provincial People's Hospital, Xi'an, China; ^2^Department of Cardiology, Beijing Hospital, National Center of Gerontology, Institute of Geriatric Medicine, Chinese Academy of Medical Sciences, Beijing, China; ^3^Graduate School, Peking Union Medical College, Chinese Academy of Medical Science, Beijing, China; ^4^Department of Cardiology, Shaanxi Provincial People's Hospital, Xi'an, China; ^5^Cardiovascular Research Center, Shaanxi Provincial People's Hospital, Xi'an, China

## Abstract

**Objective:**

Advanced glycation end products (AGEs) are featured metabolites associated with diabetic cardiomyopathy which is characterized by heart failure caused by myocyte apoptosis. Selenium was proved cardioprotective. This study was aimed at investigating the therapeutic effects and underlying mechanisms of selenium supplementation on AGE-induced heart failure.

**Methods:**

Rats and primary myocytes were exposed to AGEs. Selenium supplementation was administrated. Cardiac functions and myocyte apoptosis were evaluated. Oxidative stress was assessed by total antioxidant capacity (TAC), reactive oxygen species (ROS) generation, and GPX activity. Expression levels of DNA methyltransferases (DNMTs) and glutathione peroxidase 1 (GPX1) were evaluated. DNA methylation of the GPX1 promoter was analyzed.

**Results:**

AGE exposure elevated intracellular ROS generation, induced myocyte apoptosis, and impaired cardiac functions. AGE exposure increased DNMT1 and DNMT2 expression, leading to the reduction of GPX1 expression and activity in the heart. Selenium supplementation decreased DNMT2 expression, recovered GPX1 expression and activity, and alleviated intracellular ROS generation and myocyte apoptosis, resulting in cardiac function recovery. DNA methylation analysis in primary myocytes indicated that selenium supplementation or DNMT inhibitor AZA treatment reduced DNA methylation of the GPX1 gene promoter. Selenium supplementation and AZA administration showed synergic inhibitory effect on GPX1 gene promoter methylation.

**Conclusions:**

Selenium supplementation showed cardioprotective effects on AGE-induced heart failure by suppressing ROS-mediated myocyte apoptosis. Selenium supplementation suppressed ROS generation by increasing GPX1 expression via inhibiting DNMT2-induced GPX1 gene promoter DNA methylation in myocytes exposed to AGEs.

## 1. Introduction

The prevalence of Western diet and stationary lifestyle has been making incidence of type 2 diabetes mellitus (T2DM) increase rapidly in recent decades globally. Cardiovascular complications contributed to the major morbidity and mortality of T2DM. Diabetic cardiomyopathy (DbCM) is among these complications, which is characterized by ventricular systolic and diastolic dysfunctions [1]. Although not completely clear, according to our and other previous investigations, myocyte apoptosis which was responsible for contractile unit lost was proposed as the fundamental mechanism of DbCM [2, 3].

Advanced glycation end products (AGEs) are identical toxic metabolites largely produced in T2DM. Results from previous studies indicated that the accumulation of AGEs in diabetic hearts was sped up compared with that of nondiabetic cardiac tissue [4, 5]. It has been showed in several in vitro studies that AGEs induced apoptosis of H9c2 cells [6, 7]. According to our previous study, AGEs trigger intracellular ROS production after binding to the receptor for AGEs (RAGEs) [8]. Thus, it is reasonable to believe that AGEs are participating in inducing DbCM by promoting ROS-mediated myocyte apoptosis.

Selenium is an important trace element showing protective effects against oxidative stress-induced cell damage [9]. It was reported that selenium deficiency resulted in several cardiovascular diseases [10, 11]. Selenium is highly associated with several intracellular antioxidant proteins such as thioredoxin reductase, selenoprotein P, and glutathione peroxidase (GPX) which are critical in cell survival by maintaining oxidant-antioxidant balance [12–14]. In our previous study, we found that selenium supplementation resulted in the elevation of GPX expression and activity [15]. However, the specific molecular mechanisms were still vague. A number of investigations have reported that the selenium status showed significant impacts on gene-specific DNA methylation by affecting expression and activities of DNA methyltransferases (DNMTs) [16, 17]. Thus, we hypothesize that selenium supplementation could increase GPX1 expression by regulating DNA methylation of its promoter.

In this study, we investigated the effects of selenium supplementation on DbCM by employing an AGE-induced cardiac dysfunction animal model. We further investigated whether selenium supplementation could suppress gene promoter DNA methylation to upregulate GPX1 which suppresses AGE-induced ROS-mediated myocyte apoptosis.

## 2. Materials and Methods

### 2.1. AGE-Bovine Serum Albumin (BSA) Preparation

AGE-BSA was prepared by following previous descriptions reported by us and others [18, 19]. Glyceraidehyde (0.1 mmol/L, Sigma-Aldrich) was incubated with BSA (10 mg/mL, Gibco) in NaPO_4_ buffer solution (pH = 7.4) for 7 consecutive days at 37°C under sterilized condition. Unincorporated sugars were eliminated by using PD-10 desalting columns (GE Healthcare). Nonglycated BSA was prepared without glyceraidehyde.

### 2.2. Animals and Treatments

The protocol of establishing AGE-BSA-induced cardiac dysfunction animal model was adopted from our previous study [19]. Six-week-old Sprague-Dawley (SD) rats purchased from Vital River (Beijing, China) were accommodated for 1 week before experiments. Rats were maintained in independent polypropylene cages in a controlled artificial environment providing 25°C temperature, 50% humidity, and 12 h light-dark circle. Rats were free to chow and clean tap water. The chow contained adequate selenium (0.1 mg selenium/kg chow). AGE-BSA was administrated to rats by intraperitoneal injections at 1 mg/d for 20 consecutive days. Rats that received nonglycated BSA injections were treated as control. After AGE exposure, selenium was supplemented by intraperitoneal injections of a selenium compound sodium selenite at 0.05 mg/kg body weight for 21 consecutive days [20]. The animal study protocols were reviewed by the Institutional Ethics Committee for Animal Use and Experiment of Shaanxi Provincial People's Hospital.

### 2.3. Cardiac Function Evaluation

An invasive hemodynamic method adopted from our previous study was used to evaluate cardiac function [19]. Briefly, animals were anesthetized by isoflurane inhalation. A 3-Fench catheter (BD) was inserted into the right carotid artery. The distal end of the catheter was inserted into the left ventricle. The proximal end was connected to a pressure transducer. The PowerLab Biological Analysis System (AD Instruments) was used to plot the pressure curves. Left ventricular diastolic pressure (LVDP) and left ventricular systolic pressure (LVSP) were calculated.

### 2.4. Plasma Brain Natriuretic Peptide (BNP) Assay

The protocol of BNP assay was in accordance with our previous study [20]. Blood samples were collected via inserted catheters after cardiac function evaluations using EDTA blood collection tubes. After 1500 rpm centrifugation for 20 min, the supernatants were subjected to Triage BNP assay (Biosite).

### 2.5. Selenium Concentration Detection

Harvested blood and minced cardiac tissue samples were wet washed with mixed acids (perchloric acid and nitric acid) in reaction tubes. Excess acids were removed and samples were titrated to 10 mL with pure water and then subjected to selenium concentration detection. The detection was based on flameless atomic absorption spectrophotometry by using a Z5000 spectrophotometer (Hitachi) with selenium cathode lamp. Standard curves were plotted to calculate the selenium concentrations.

### 2.6. Primary Myocyte Isolation and Treatments

Primary myocytes were isolated from neonatal SD rats. Liberase (4.5 mg/mL, Roche) was used to perfuse the harvested heart under sterile condition. Primary myocytes were isolated and purified in accordance with protocols of our and other previous studies [19, 21]. Cardiomyocytes were cultured in minimum essential medium (MEM) (Gibco) supplemented with BSA (10%, Gibco), 2,3-butanedionemonoxime (10 mmol/L, Sigma-Aldrich), L-glutamine (2 mmol/L, Gibco), and antibiotic-antimycotic agent (10,000 U/mL penicillin, 25 *μ*g/mL amphotericin B, and 10,000 *μ*g/mL streptomycin, Gibco) at 37°C in an incubator providing a humidified atmosphere containing 5% CO_2_ and 95% fresh air. Myocytes were treated with AGE-BSA at 100 *μ*g/mL for 24 h. Several cells were pretreated with sodium selenite at 10 *μ*mol/L and/or 5-aza-2′-deoxycytidine (AZA) (Sigma-Aldrich) at 500 nmol/L for 24 h. The exposure concentration of sodium selenite solution was decided by our pilot CCK-8 assay (TableS1).

### 2.7. Cell Apoptosis Assessment

Cell apoptosis *in vivo* was detected by terminal transferase UTP nick end labeling (TUNEL). Harvested and perfused hearts were trimmed and embedded by optimal cutting temperature compound (OCT, Sakura), which were further made into 5 *μ*m thick sections. A Step-One Apoptosis Assay Kit (Beyotime) was applied to the slides according to the manufacturer's instructions. DAPI (Abcam) was used to stain the nuclei. Cell apoptosis *in vitro* was detected by flow cytometry. Suspended myocytes were incubated with binding buffer, PI (Invitrogen), and FITC (Invitrogen) in the dark for 15 min. A flow cytometer (FACSCalibur, BD) was used to analyze the apoptosis.

### 2.8. GPX Enzymatic Activity Assessment

The GPX activity was assessed with a colorimetric method. After being washed by PBS, harvested cardiac tissue homogenate or myocytes were processed by the RIPA lysis buffer system (Santa Cruz) and cell extracts were acquired. A Glutathione Peroxidase Assay Kit (Nanjing Jiancheng Bioengineering Institute) was used to determine the enzymatic activities. Assays were carried out according to the manufacturer's instructions.

### 2.9. *In Situ* ROS Detection and Total Antioxidant Capacity (TAC) Assay

*In situ* ROS generation was detected by 2,7-dichlorofluorescein diacetate (DCFH-DA) stains. Fresh cardiac cryostat sections and myocyte suspension were incubated with DCFH-DA (Molecular Probes) in a dark incubator at 37°C for 30 min. Samples were excited at 488 nm and measured at 530 nm. Sections were observed with an inverted fluorescence microscope, and cell suspension was analyzed with a flow cytometer (FACSCalibur, BD). The total antioxidant capacity of cell and tissue homogenates was assessed with a T-AOC Assay Kit (Beyotime) by following the instructions provided by the manufacturer.

### 2.10. *GPX1* DNA Promoter Methylation Evaluation

The protocol was in accordance with our previous investigation [22]. DNA was extracted from prepared myocytes with a DNA Extraction Kit (TianGen). Bisulfate chemical modification of DNA was carried out by using the EZ DNA Methylation-Gold Kit (Zymo). The primer of *GPX1* promoter methylation was designed and synthesized by GeneChem (Shanghai, China). The sequence was as follows: forward: 5′-TTTGAAGAAGGTAGAGATATGGTAAATAG-3′ and reverse: 5′- ACAAAACTTCACAAAATAAAACACC-3′. After denaturing at 95°C for 5 min, each reaction was carried out with a touchdown PCR protocol for 10 cycles. The annealing and extension temperatures were 60°C and 72°C, respectively. PCR products were purified by gel electrophoresis separation (Promega) and linked to PMD19-T vectors (TaKaRa). DNA was extracted and sequenced after amplification and identification.

### 2.11. Real-Time Quantitative Polymerase Chain Reaction (RT-PCR)

RNA from myocytes and cardiac tissue were extracted by TRIzol Reagent (Invitrogen) by following the instructions provided by the manufacturer. Reverse transcription was accomplished by using the PrimeScript™ RT Reagent Kit with a gDNA Eraser (TaKaRa). The purity and concentration of cDNA were evaluated by NanoDrop Spectrophotometer (Thermo). PCR was performed by using TB Green® Premix Ex Taq™ II (TaKaRa) and ABI Prism Step-One Plus Detection System (Applied Byosystems). Primers for *GPX1*, *DNMT1*, *DNMT2*, *DNMT3a*, *DNMT3b*, and *GAPDH* were listed in Table 1. Relative expression levels of mRNAs were calculated by the 2^−ΔΔ*Ct*^ method when GAPDH was introduced as the internal reference.

### 2.12. Western Blotting

Minced cardiac ventricular tissue and harvested myocytes were homogenized and lysed with the RIPA lysis buffer system (Santa Cruz) supplemented with PMSF (100 mol/L, Santa Cruz) on ice. Protein was extracted with a Total Protein Extraction Kit (Beyotime). After the concentration was detected with a BCA Kit (Beyotime), protein samples were subjected to and separated by 8% or 12% sodium dodecyl sulfate polyacrylamide gel electrophoresis (SDS-PAGE) and then transferred electronically to polyvinylidene fluoride (PVDF) membranes. After incubation with blocking buffer (Beyotime) for 15 min, the membranes were then incubated with primary antibodies against GPX1 (1 : 1000, Abcam), DNMT1 (1 : 1000, CST), DNMT2 (1 : 1000, Abcam), DNMT3a (1 : 1000, CST), DNMT3b (1 : 1000, CST), caspase3 (caspase3, 1 : 2000, Abcam), and GAPDH (1 : 1000, Abcam) at 4°C for 10 h. Membranes were washed by TBST and then incubated with HRP-conjugated secondary antibodies (1 : 10000, CST) at 20°C for 2 h. After TBST washing, an ECL Kit (Beyotime) was used to develop the membranes which were then exposed on X-ray films. Intensities of immunoblots were analyzed by ImageJ software.

### 2.13. Statistics

In the present study, acquired data were presented in (mean ± standard deviations) or percentage manners. Comparisons of differences between two groups and multiple groups were carried out by *t*-tests and one-way ANOVA. The NSK test was carried out as the post hoc test. Compared differences were considered statistically significant when *P* < 0.05. All analysis was performed by using PASW Statistics software (version 20.0).

## 3. Results

### 3.1. Selenium Supplementation Increased Selenium Contents in Circulation and Cardiac Tissue

Determination results of selenium contents in blood and cardiac tissue samples were demonstrated in Table 2. Significant selenium content elevations were observed in blood and cardiac tissue collected from rats injected with sodium selenite.

### 3.2. Selenium Supplementation Improved Cardiac Systolic and Diastolic Functions by Reducing Myocyte Apoptosis in AGE-Exposed Rats

**Figure 1:**
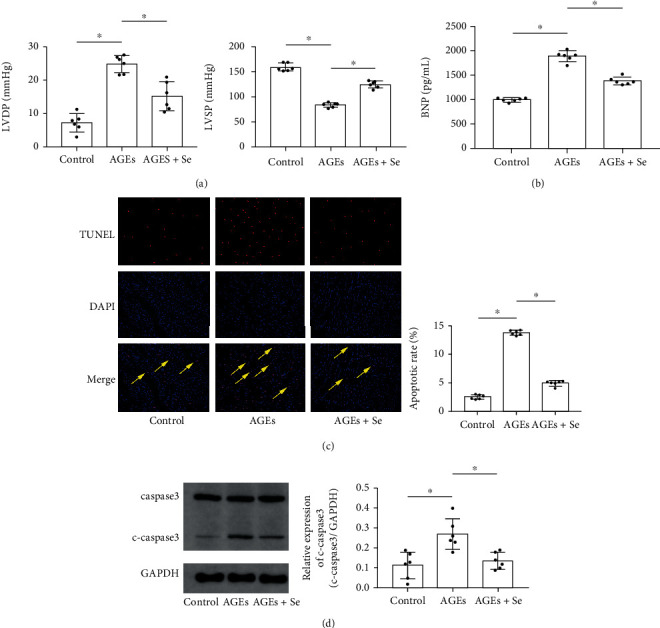
(a) Columns indicated the calculated left ventricular systolic pressure (LVSP) and left ventricular diastolic pressure (LVDP) in rats in control, AGE, and AGE + Se groups. (b) Columns indicated the detected BNP concentrations in blood samples collected from rats in control, AGE, and AGE + Se groups. (c) Captured fluorescent images of TUNEL assay of cardiac tissue. TUNEL-positive cells were tagged with red fluorescence and pointed by yellow arrows. Columns indicated the apoptosis rate in cardiac tissue harvested from rats in control, AGE, and AGE + Se groups, respectively. (d) Immunoblots of caspase3, cleaved caspase3 (c-caspase3), and GAPDH were demonstrated. Columns indicated relative expression levels of c-caspase3 in cardiac tissue harvested from rats in control, AGE, and AGE + Se groups, respectively. Magnification of images in Figure 1(a) was 200 (control: rats treated with control BSA; AGEs: rats treated with AGE-BSA; AGEs + Se: rats treated with AGE-BSA then supplemented with sodium selenite (*n* = 6, ^∗^*P* < 0.05)).

As demonstrated in Figures 1(a) and 1(b), AGE exposure impaired cardiac functions which were evidenced by dramatically decreased LVSP and increased LVDP and plasma BNP concentrations. The selenium supplementation significantly improved LVDP, LVSP, and decreased BNP concentration in AGE-exposed rats. As demonstrated in Figures 1(c) and 1(d), compared with control, AGEs exposure dramatically increased myocyte apoptosis which was evidenced by TUNEL assay and increased c-caspase3 expression which was evidenced by Western blotting. The selenium supplementation, however, inhibited myocyte apoptosis in AGE-exposed hearts.

### 3.3. Selenium Supplementation Alleviated Cardiac Oxidative Stress by Reducing ROS Generation, Restoring GPX1, and Recovering TAC in AGE-Exposed Animals

**Figure 2:**
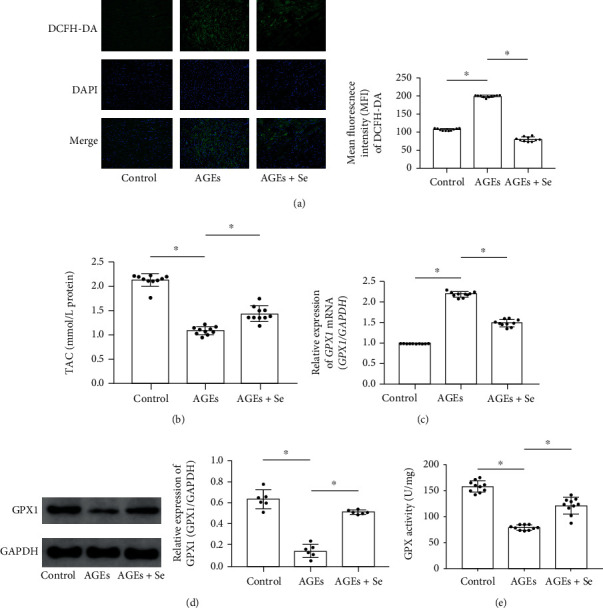
(a) Images of ROS indicator DCFH-DA stains of cardiac tissue sections were demonstrated. Columns indicated mean fluorescent intensities of DCFH-DA in control, AGE, AGE + Se groups, respectively. (b) Columns indicated the detected total antioxidant capacity (TAC) of cardiac tissue homogenates from rats in control, AGE, and AGE + Se groups. (c) Columns indicated relative expression levels of *GPX1* mRNA in cardiac tissue of rats from control, AGE, and AGE + Se groups. (d) Immunoblots of GPX1 and GAPDH were demonstrated. Columns indicated relative expression of GPX1. (e) Columns indicated measured GPX activity cardiac tissue homogenates from rats in control, AGE, and AGE + Se groups. Magnification of images in Figure 2(a) was 200 (control: rats treated with control BSA; AGEs: rats treated with AGE-BSA; AGEs + Se: rats treated with AGE-BSA then supplemented with sodium selenite (*n* = 10, ^∗^*P* < 0.05)).

Compared with control, AGE exposure increased ROS generation (Figure 2(a)) and decreased TAC (Figure 2(b)) and GPX1 expression (Figures 2(c) and 2(d)) and activity (Figure 2(e)) in hearts harvested from AGE-exposed rats. The selenium supplementation dramatically inhibited ROS generation (Figure 2(a)) and restored TAC (Figure 2(b)) and GPX1 expression (Figures 2(c) and 2(d)) and activity (Figure 2(e)) in cardiac tissue collected from AGE-exposed rats.

### 3.4. Selenium Supplementation and AZA Treatment Suppressed AGE-Induced Myocyte Oxidative Stress and Apoptosis *In Vitro*

**Figure 3:**
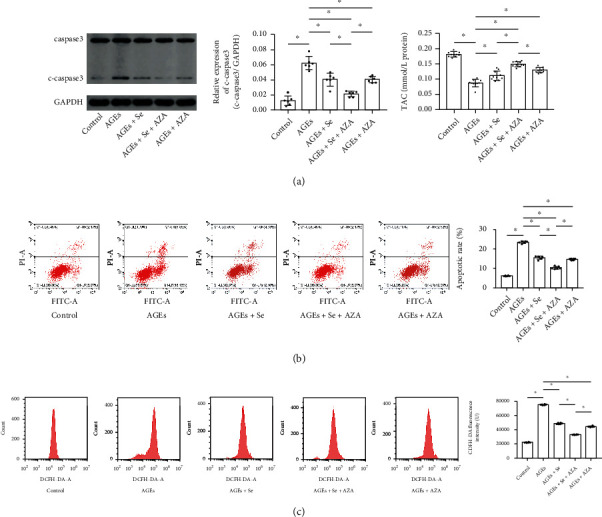
(a) Immunoblots of caspase3, cleaved caspase3 (c-caspase3), and GAPDH were demonstrated. Columns indicated relative expression levels of c-caspase3 in myocytes from control, AGEs, AGE + Se, AGE + Se + AZA, and AGE + AZA groups. (b) Plotted charts of flow cytometry detecting apoptotic myocytes were demonstrated. Columns indicated the apoptotic rate of myocytes from control, AGE, AGE + Se, AGE + Se + AZA, and AGE + AZA groups. (c) Plotted charts of flow cytometry measuring DCFH-DA fluorescence in myocytes were demonstrated. Columns indicated fluorescent intensities of DCFH-DA in myocytes from control, AGE, AGE + Se, AGE + Se + AZA, and AGE + AZA groups (control: myocytes treated with control BSA; AGEs: myocytes incubated with AGEs; AGEs + Se: AGE-exposed myocytes treated with sodium selenite; AGEs + Se + AZA: AGE-exposed myocytes treated with sodium selenite and AZA; AGEs + AZA: AGE-exposed myocytes treated with AZA (*n* = 10, ^∗^*P* < 0.05)).

AGE-exposed myocytes received selenium supplementation and/or AZA treatments. As demonstrated in Figures 3(a)–3(c), evidenced by caspase3 expression, flowcytometry, and T-AOC assay, selenium supplementation or AZA treatment significantly suppressed cell apoptosis and intracellular ROS generation and restored TAC in AGE-treated myocytes. Moreover, selenium supplementation with AZA treatment showed stronger inhibitory effects on oxidation and apoptosis in myocytes exposed to AGEs.

### 3.5. Selenium Supplementation and AZA Treatment Altered AGE-Induced DNMT Expression Changes *In Vivo* and *In Vitro*

**Figure 4:**
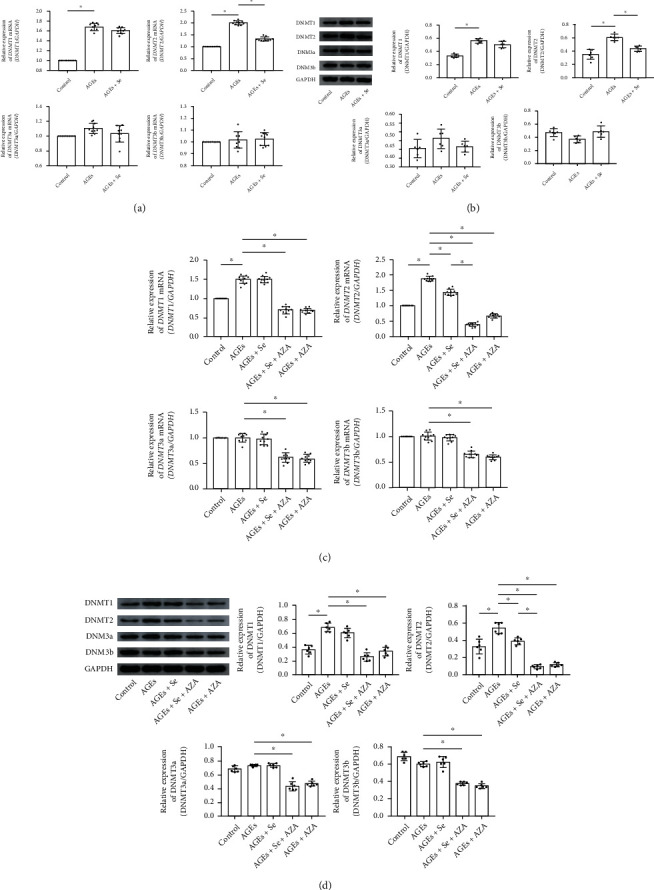
(a) Columns indicated relative mRNA expression levels of *DNMT1*, *DNMT2*, *DNMT3a*, and *DNMT3b* in cardiac tissue of rats from control, AGE, and AGE + Se groups. (b) Immunoblots of DNMT1, DNMT2, DNMT3a, and DNMT3b. Columns indicated relative expression levels of DNMT1, DNMT2, DNMT3a, and DNMT3b in cardiac tissue of rats from control, AGE, and AGE + Se groups (control: rats treated with control BSA; AGEs: rats treated with AGE-BSA; AGEs + Se: rats treated with AGE-BSA then supplemented with sodium selenite (*n* = 6 or 10, ^∗^*P* < 0.05)). (c) Columns indicated relative mRNA expression levels of *DNMT1*, *DNMT2*, *DNMT3a*, and *DNMT3b* in myocytes from control, AGEs, AGE + Se, AGE + Se + AZA, and AGE + AZA groups, respectively. (d) Immunoblots of DNMT1, DNMT2, DNMT3a, and DNMT3b. Columns indicated relative expression levels of DNMT1, DNMT2, DNMT3a, and DNMT3b in myocytes from control, AGEs, AGE + Se, AGE + Se + AZA, and AGE + AZA groups, respectively (control: myocytes treated with control BSA; AGEs: myocytes incubated with AGEs; AGEs + Se: AGE-exposed myocytes treated with sodium selenite; AGEs + Se + AZA: AGE-exposed myocytes treated with sodium selenite and AZA; AGEs + AZA: AGE-exposed myocytes treated with AZA (*n* = 6 or 10, ^∗^*P* < 0.05)).

Both RT-PCR and Western blotting were employed to evaluate expression levels of DNMTs *in vivo* and *in vitro*. As demonstrated in Figures 4(a) and 4(b), compared with control, expression levels of DNMT1 and DNMT2, rather than DNMT3a and DNMT3b, increased significantly in cardiac tissue harvested from AGE-exposed rats. Selenium supplementation significantly lowered expression levels of DNMT2 rather than other DNMTs in AGE-exposed hearts. The *in vitro* results were demonstrated in Figures 4(c) and 4(d). AGE exposure significantly increased expression levels of DNMT1 and DNMT2, rather than DNMT3a and DNMT3b in primary myocytes. AZA treatment decreased expression levels of all DNMTs. Selenium supplementation decreased the expression level of DNMT2 rather than other DNMTs in AGE-exposed primary myocytes. Moreover, selenium supplementation and AZA treatment showed synergic inhibitory effect on DNMT2 expression in AGE-exposed primary myocytes.

### 3.6. Selenium Supplementation and AZA Treatment Altered DNA Methylation of the *GPX1* Promoter, GPX1 Expression, and Activity in AGE-Exposed Myocytes

**Figure 5:**
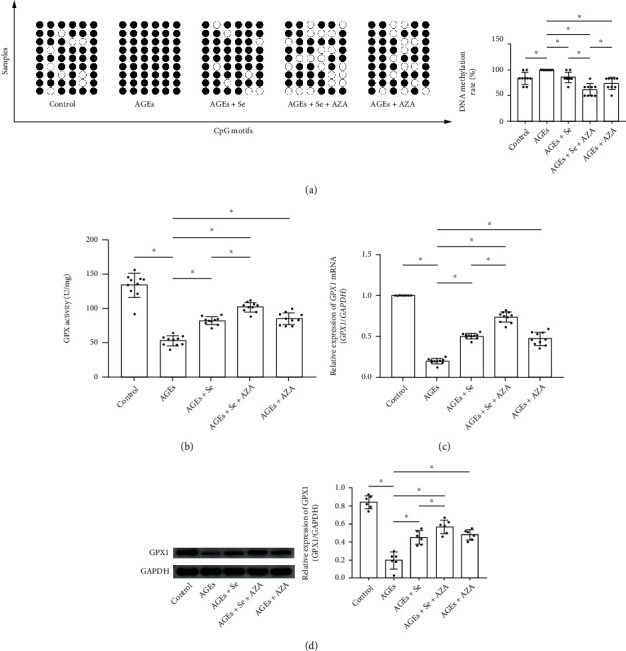
(a) Diagram of DNA methylation of the *GPX1* gene promoter was plotted. Black dots indicated methylated sites while white dots indicated demethylated sites. Columns indicated the methylation rate of the *GPX1* gene promoter in myocytes from control, AGE, AGE + Se, AGEs+Se + AZA, and AGE + AZA groups (control: myocytes treated with control BSA; AGEs: myocytes incubated with AGEs; AGEs + Se: AGE-exposed myocytes treated with sodium selenite; AGEs + Se + AZA: AGE-exposed myocytes treated with sodium selenite and AZA; AGEs + AZA: AGE-exposed myocytes treated with AZA (*n* = 9, ^∗^*P* < 0.05)); (b) columns indicated the GPX activity in myocytes from control, AGE, AGE + Se, AGE + Se + AZA, and AGE + AZA groups. (c) Columns indicated relative expression levels of *GPX1* mRNA in myocytes from control, AGEs, AGE + Se, AGE + Se + AZA, and AGE + AZA groups. (d) Immunoblots of GPX1 and GAPDH were demonstrated. Columns indicated relative expression levels of GPX1 in myocytes from control, AGE, AGE + Se, AGE + Se + AZA, and AGE + AZA groups (control: myocytes treated with control BSA; AGEs: myocytes incubated with AGEs; AGEs + Se: AGE-exposed myocytes treated with sodium selenite; AGEs + Se + AZA: AGE-exposed myocytes treated with sodium selenite and AZA; AGEs + AZA: AGE-exposed myocytes treated with AZA (*n* = 6 or 10, ^∗^*P* < 0.05)).

As demonstrated in Figure 5(a), according to bisulfate sequencing DNA methylation analysis, six methylation sites of the *GPX1* promoter were spotted. The *GPX1* promoter DNA methylation rate was increased in AGE-exposed myocytes (100%) compared with control (83.33%). Selenium supplementation lowered the methylation rate to 85.19%. AZA treatment lowered the methylation rate to 74.08%. Selenium supplementation and AZA treatment showed synergic inhibition on *GPX1* promoter DNA methylation, which reduced the methylation rate to 61.11%. The specific CpG motif changes of the methylation sites were demonstrated in TableS2. Both activity (Figure 5(b)) and expression of GPX1 (Figures 5(c) and 5(d)) decreased significantly in AGE-exposed myocyte compared with control. The selenium supplementation or AZA treatment restored GPX1 expression and activity. Moreover, selenium and AZA showed synergic effect in restoring GPX1 expression and activity in myocytes exposed to AGEs.

## 4. Discussion

In the present investigation, both *in vivo* and *in vitro* studies were performed to evaluate the effects of selenium supplementation on AGE-induced cardiac dysfunction. Involved underlying mechanisms were also studied. Our data suggested that by inhibiting oxidative stress-mediated myocyte apoptosis, selenium supplementation significantly improved AGE-induced cardiac systolic and diastolic dysfunctions. Moreover, results from our study proposed that selenium supplementation suppressed cardiac oxidative stress by restoring antioxidant-oxidant balance via increasing GPX1. Further analysis indicated that selenium supplementation facilitated GPX1 gene promoter DNA demethylation by reducing DNMT2.

AGEs are characterized pathological metabolites of T2DM, which are especially massively fostered under condition of uncontrolled and sustained hyperglycemia. It was believed that AGE-induced cell damages were highly associated with diabetic cardiovascular complications. Glycemic control improved LV mechanical performance and outcomes of enrolled subjects with T2DM [23]. According to our and other previous investigations, AGE injections caused significant cardiac systolic and diastolic dysfunctions [19, 24]. It has been well accepted that cardiac apoptosis-caused contractile unit loss is one of the major contributors to cardiac dysfunctions and features of failing hearts [25, 26].

Oxidative stress is proposed as the connection between AGEs and cardiac muscle apoptosis. By interacting with receptors for RAGEs, AGEs induced intracellular ROS production via nicotinamide adenine dinucleotide phosphate (NADPH) oxidase systems, which was confirmed and testified by other and our investigations [8, 27]. Excessive ROS would break the antioxidant-oxidant balance, triggering cell apoptosis by multiple mechanisms such as mitochondria and endoplasmic reticulum stress-dependent pathways [28, 29]. In this study, we found that AGE administration facilitated ROS production in myocytes which further activate apoptotic events. As a result, due to contractile unit loss, both cardiac systolic and diastolic functions were impaired.

In mammals, there are eight subtypes of GPXs which belong to a family of phylogenetically related enzymes. GPX1 is a typical selenoprotein due to localization of a selenocysteine in the catalytic center. GPX1 is capable of reducing organic hydroperoxides to water or alcohols by using glutathione (GSH) as a reductant. GPX1 deficiency would lead to impairment of the antioxidant defense system, resulting in cell dysfunctions and apoptosis. Previous investigations reported a significant reduction of GPX1 levels in patients with T2DM [30].

Epigenetic changes such as DNA methylation take place and play critical roles in pathophysiological processes of T2DM. DNA methylation occurs at CpG islands located in noncoding regions and is mainly performed by DNMTs which were reported dysregulated under the circumstance of T2DM according to our and other previous studies [22]. In this study, we investigated expression changes of DNMTs in hearts and primary myocytes exposed to AGEs. The results suggested that AGE exposure increased expression levels of DNMT1 and DNMT2 *in vivo* and *in vitro*. Our further in-depth analysis results indicated that AGE exposure dramatically facilitated DNA methylation of the *GPX1* gene promoter, leading to the inhibition of GPX1 expression in myocytes. As a result, the intracellular antioxidant-oxidant balance was broken and apoptosis of myocytes was thus induced. Administration of global DNMT inhibitor AZA decreased the DNA methylation level of the *GPX1* gene promoter, leading to GPX1 expression increasing which restored the antioxidant defense system. As a result, myocyte apoptosis was reduced and cardiac functions were recovered.

Selenium is a very important biological trace element with various beneficial effects. The link between selenium and GPX1 has been established in investigating the selenium content in ground drinking water and Keshan disease [31, 32]. Selenium deficiency induced obvious oxidative stress-mediated cell damage which resulted from excessive ROS production [33, 34]. Supplemented selenium strengthened antioxidant capabilities by incorporating into antioxidant enzymes. Our and other previous investigations indicated that the effect of selenium supplementation reduced the cardiac ROS level and improved cardiac functions in different heart failure models. [20, 35]. Results from several population studies suggested the inhibitory effects of selenium supplementation on hypermethylation of certain disease-related genes [36, 37]. In the present study, we found that selenium supplementation showed significant suppressive effects on DNMT2 and further relieved the DNA hypermethylation status of the *GPX1* gene promoter. AZA enhanced the inhibitory effects of selenium supplementation on DNMT2, leading to elevation of GPX1 expression and activity. As a result, myocyte apoptosis was suppressed and cardiac functions were improved in AGE-exposed rats.

In summary, our results suggested that selenium supplementation improved AGE-induced cardiac dysfunctions by impacting epigenetics of myocytes. Specifically, selenium inhibited DNMT2 expression and further reduced *GPX1* promoter DNA methylation. Increased GPX1 restored antioxidant-oxidant balance to prevent myocytes from apoptosis. We believe that results from this study would add more knowledge to our current understanding concerning the mechanisms of DbCM. Selenium supplementation at appropriate dosage would be a promising strategy in the treatment of DbCM. More in-depth preclinical and clinical trials would be needed.

## Figures and Tables

**Table 1:** Primer sequence for RT-PCR.

Gene	Primer	Sequence (5′-3′)	PCR products
*GAPDH*	Forward	ACAGCAACAGGGTGGTGGAC	253 bp
	Reverse	TTTGAGGGTGCAGCGAACTT	

*GPX1*	Forward	GACCGACCCCAAGTACATCA	155 bp
	Reverse	GCAGGGCTTCTATATCGGGT	

*DNMT1*	Forward	GTGTTTTCTGGCTGTGGAGG	235 bp
	Reverse	CACACAGCATCTCCACATCG	

*DNMT2*	Forward	ACAAGAGGGCTGCTGATACA	238 bp
	Reverse	TCACTCTCAGCTTGCCTTCT	

*DNMT3a*	Forward	GGTGCTTTTGTGTGGAGTGT	165 bp
	Reverse	TGGCGAAGAACATCTGGAGT	

*DNMT3b*	Forward	GAGGGGAAGATGAGGAGAGC	197 bp
	Reverse	CTGATAGCCGTCCTCATCGT	

**Table 2:** Selenium contents in blood and cardiac tissue.

Groups	Selenium (blood), mg/L	Selenium (tissue), mg/kg wet tissue
Control	0.128 ± 0.012	0.334 ± 0.021
AGEs	0.123 ± 0.009	0.335 ± 0.022
AGEs + Se	0.293 ± 0.025^∗,∗∗^	0.489 ± 0.016^∗,∗∗^

Control: rats treated with control BSA; AGEs: rats treated with AGE-BSA; AGEs + Se: rats treated with AGE-BSA and then supplemented with sodium selenite (*n* = 10, ^∗^significantly different compared with control, *P* < 0.05; ^∗∗^significantly different compared with AGEs, *P* < 0.05).

## Data Availability

Original data would be available upon reasonable requests.

## Supplementary Materials

Supplementary Materials TableS1: pilot CCK-8 assay determining optimistic sodium selenite concentration. TableS2: analysis of methylation sites and methylation rate of GPX1 DNA promoter in primary myocytes (*n* = 9)
