# Supplementary material for: Selenium Supplementation Improved Cardiac Functions by Suppressing DNMT2-Mediated GPX1 Promoter DNA Methylation in AGE-Induced Heart Failure
Source: Oxid Med Cell Longev. 2022 Apr 6;2022:5402997. doi: 10.1155/2022/5402997 (PMC9007686; doi:10.1155/2022/5402997)
Supplement: Supplementary Materials — TableS1: pilot CCK-8 assay determining optimistic sodium selenite concentration. TableS2: analysis of methylation sites and methylation rate of GPX1 DNA promoter in primary myocytes (n = 9) [file 5402997.f1.docx]

**Contents**

**Supplemental Tables**

**TableS1**

Pilot CCK-8 assay determining optimistic sodium selenite concentration

| Treatments | Test1 | Test2 | Test3 | Proliferation rate (%) |
| --- | --- | --- | --- | --- |
| Blank | 0.163 | 0.166 | 0.167 | - |
| Control | 0.893 | 0.838 | 0.845 | 100.0 |
| AGEs | 0.611 | 0.614 | 0.602 | 64.0 |
| AGEs+Se (1μmmol/L) | 0.656 | 0.671 | 0.661 | 71.7 |
| AGEs+Se (5μmmol/L) | 0.703 | 0.757 | 0.734 | 81.6 |
| AGEs+Se (10μmmol/L) | 0.735 | 0.771 | 0.785 | 86.3 |
| AGEs+Se (20μmmol/L) | 0.753 | 0.744 | 0.735 | 83.4 |
| AGEs+Se (40μmmol/L) | 0.703 | 0.717 | 0.696 | 77.9 |
| AGEs+Se (80μmmol/L) | 0.602 | 0.628 | 0.617 | 65.0 |
| AGEs+Se (150μmmol/L) | 0.426 | 0.454 | 0.455 | 40.3 |
| AGEs+Se (300μmmol/L) | 0.376 | 0.337 | 0.350 | 26.6 |

control: rats treated with control BSA; AGEs: rats treated with AGEs-BSA; AGEs+Se: rats treated with AGEs-BSA thensupplemented with sodium selenite

**TableS2**

Analysis of methylation sites and methylation rate of GPX1 DNA promoter in primary myocytes (n=9)

| Methylation sites | 1 | 2 | 3 | 4 | 5 | 6 | Methylation sites number | Methylation rate% |
| --- | --- | --- | --- | --- | --- | --- | --- | --- |
| Anticipated | CG | CG | CG | CG | CG | CG |  |  |
| Control | CG | CG | CG | CG | CG | CG | 6 | 100 |
|  | CG | CG | TG | CG | CG | CG | 5 | 83.33 |
|  | CG | CG | CG | TG | TG | CG | 4 | 66.67 |
|  | CG | TG | CG | CG | CG | CG | 5 | 83.33 |
|  | CG | CG | CG | CG | CG | CG | 6 | 100 |
|  | CG | TG | CG | CG | CG | CG | 5 | 83.33 |
|  | CG | CG | TG | CG | TG | CG | 4 | 66.67 |
|  | CG | CG | CG | CG | TG | CG | 5 | 83.33 |
|  | CG | TG | CG | CG | CG | CG | 5 | 83.33 |
| AGEs | CG | CG | CG | CG | CG | CG | 6 | 100 |
|  | CG | CG | CG | CG | CG | CG | 6 | 100 |
|  | CG | CG | CG | CG | CG | CG | 6 | 100 |
|  | CG | CG | CG | CG | CG | CG | 6 | 100 |
|  | CG | CG | CG | CG | CG | CG | 6 | 100 |
|  | CG | CG | CG | CG | CG | CG | 6 | 100 |
|  | CG | CG | CG | CG | CG | CG | 6 | 100 |
|  | CG | CG | CG | CG | CG | CG | 6 | 100 |
|  | CG | CG | CG | CG | CG | CG | 6 | 100 |
| AGEs+Se | CG | TG | CG | CG | CG | CG | 5 | 83.33 |
|  | CG | CG | TG | CG | CG | CG | 5 | 83.33 |
|  | CG | CG | CG | CG | CG | TG | 5 | 83.33 |
|  | CG | CG | CG | CG | CG | CG | 6 | 100 |
|  | CG | CG | CG | CG | CG | CG | 6 | 100 |
|  | CG | TG | CG | CG | CG | CG | 5 | 83.33 |
|  | CG | CG | CG | CG | TG | CG | 5 | 83.33 |
|  | CG | CG | TG | CG | CG | CG | 5 | 83.33 |
|  | CG | CG | CG | CG | TG | TG | 4 | 66.67 |
| AGEs+AZA | CG | CG | TG | TG | CG | CG | 4 | 66.67 |
|  | CG | CG | TG | CG | CG | CG | 5 | 83.33 |
|  | CG | CG | CG | TG | TG | CG | 4 | 66.67 |
|  | CG | CG | CG | TG | CG | CG | 5 | 83.33 |
|  | CG | CG | TG | CG | CG | CG | 5 | 83.33 |
|  | CG | CG | CG | TG | TG | CG | 4 | 66.67 |
|  | CG | CG | TG | CG | CG | CG | 5 | 83.33 |
|  | CG | CG | CG | CG | TG | CG | 5 | 83.33 |
|  | CG | TG | CG | TG | CG | TG | 3 | 50.00 |
| AGEs+Se+AZA | CG | CG | TG | CG | CG | TG | 4 | 66.67 |
|  | CG | CG | TG | TG | TG | CG | 3 | 50.00 |
|  | TG | CG | CG | CG | CG | CG | 5 | 83.33 |
|  | CG | CG | CG | TG | TG | CG | 4 | 66.67 |
|  | CG | TG | TG | TG | CG | CG | 3 | 50.00 |
|  | TG | CG | CG | TG | CG | TG | 3 | 50.00 |
|  | CG | CG | CG | CG | TG | TG | 4 | 66.67 |
|  | CG | CG | CG | CG | TG | TG | 4 | 66.67 |
|  | TG | TG | TG | CG | CG | CG | 3 | 50.00 |

control: rats treated with control BSA; AGEs: rats treated with AGEs-BSA; AGEs+Se: rats treated with AGEs-BSA thensupplemented with sodium selenite
